# Anaesthetic considerations of adults with Morquio's syndrome - a case report

**DOI:** 10.1186/1471-2253-10-2

**Published:** 2010-02-26

**Authors:** Anne Marie McLaughlin, Muhammad Farooq, Maria B Donnelly, Kieran Foley

**Affiliations:** 1 Department of Anaesthesia and Critical Care, Adelaide and Meath National Children Hospital Dublin, Dublin, Ireland

## Abstract

**Background:**

The anaesthetic management of patients with Morquio syndrome is complicated by a number of factors including odontoid hypoplasia, atlantoaxial instability, thoracic kyphosis, and deposition of mucopolysaccharides in the soft tissue of the oropharnyx.

**Case presentation:**

Herein we describe the anaesthetic considerations and management of a 26 year old adult with Morquio syndrome, who presented for an elective hip replacement.

**Conclusion:**

This report details an awake fiberoptic intubation in an adult with Morquio syndrome. We recommend that this approach be considered in patients with Morquio syndrome undergoing general anaesthesia.

## Background

Morquio's syndrome also known as Mucopolysaccharidosis type IV is an autosomal recessive disorder caused by deficiency of n-acetylgalactosamine-6-sulphate. Morquio in Uruguay and Brailsford in England simultaneously described this syndrome in 1929[1]. The incidence is unknown but is estimated to be between 1 in 75 000 population in Northern Ireland to 1 in 200 000 population in British Columbia [2,3]. Morquio's syndrome is characterised by a defect in the degradation of keratin sulphate resulting in the accumulation of mucopolysaccharides. At birth, a patient with Morquio's syndrome may appear healthy, however as the child grows into adulthood, various manifestations of this syndrome begin to emerge, including coarse facial features, prognathism, a broad mouth, a short nose with anteverted nares and a flat bridge, widely spaced teeth and macrocephaly. Other features include aortic valve incompetence hepatomegaly, inguinal hernias, mixed hearing loss and ocular complications including clouding of the corneas, pigmented degenerative retinal lesions or glaucoma. Pulmonary complications include a restrictive defect due to kyphoscoliosis resulting in decreased lung volumes and ventilation-perfusion mismatching and central or obstructive sleep apnoea, which can result in pulmonary hypertension and cor pulmonale. Characteristic vertebral abnormalities include anterior hypoplasia of T12, L1 or L2, which may give rise to lumbar kyphosis. Hypoplasia of the dens is a common and severe manifestation that may lead to atlantoaxial instability, compression of the cervical spinal cord, and complications during endotracheal intubation. The limb-bone abnormalities may include short diaphyses, curving of the metaphyses and poor development of the epiphyses. Pelvic abnormalities include widening of the acetabula, hypoplasia of the femoral heads, with valgus deformity of the femoral necks[4].

## Case presentation

A 26 year old man with a background of Morquio syndrome, was admitted for an elective left total hip replacement. Osteoarthritis of the hips secondary to avascular necrosis of the head of femur bilaterally had been diagnosed. There was no past medical history of note; however review of systems revealed snoring and daytime somnolence, suggestive of obstructive sleep apnoea. Clinical inspection demonstrated a man of short stature, his weight was 63 Kg and height 150 cm, with a large head and short neck, the head appeared to be sitting directly on his thorax. On inspection the thorax had an increased anterior-posterior diameter and kyphosis. Clinical examination including respiratory, neurological and cardiovascular systems were normal. Pulmonary function testing and baseline laboratory tests were normal.

**Figure 1:**
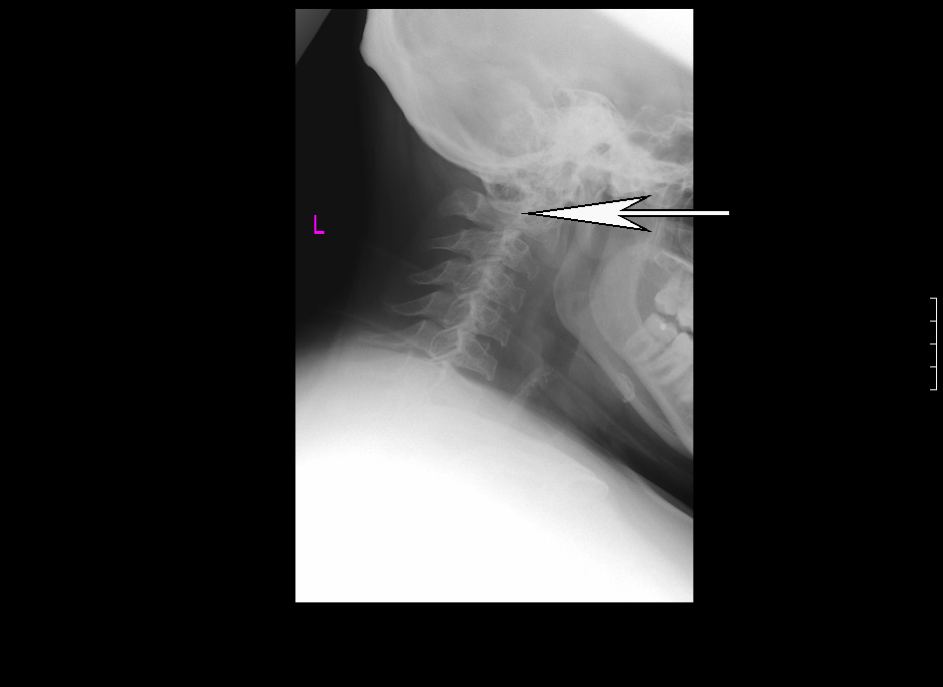
**Lateral cervical spine x-ray demonstrating loss of normal cervical lordosis, anterior beaking of the cervical bodies and hypoplasia of odontoid peg**. The atlanto axial space on flexion is 3 mm, the upper limit of normal.

**Figure 2:**
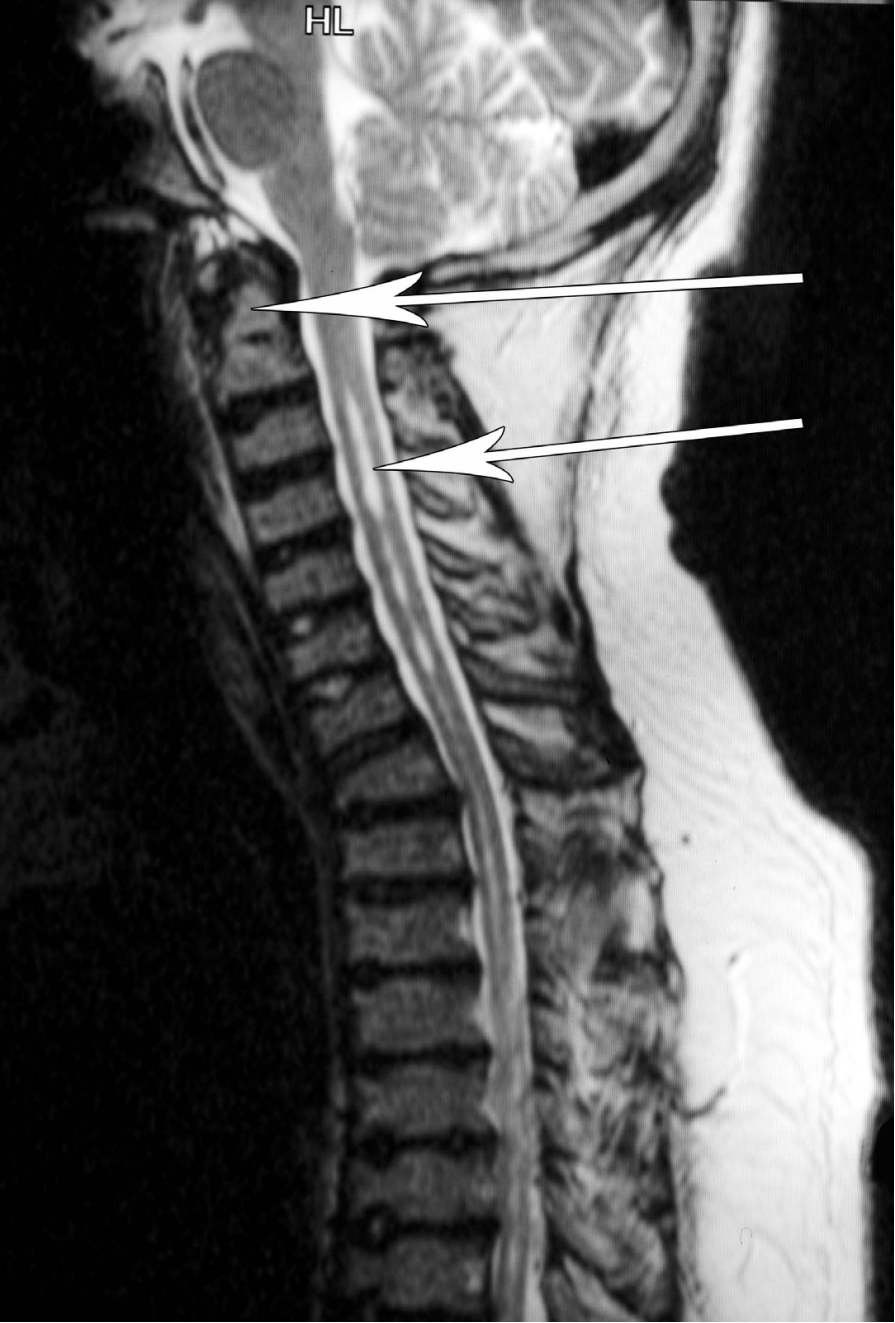
**MRI cervical spine saggital section, with saturation band, anterior beaking of the cervical vertebrae and syrinx between C3 and C7 is noted**. Arrows indicate odontoid peg and syrinx.

Preoperative airway examination demonstrated bulky soft tissue in the pharynx, grade 2 macroglossia, an enlarged uvula and tonsils, an exuberant gag reflex and a Mallampatti grade II airway. Preanaesthetic neutral and extension cervical x-rays were performed and demonstrated a hypoplastic odontoid process, and widening of the atlanto axial joint upon flexion, the joint measured 3 mm in diameter, the upper limit of normal (figure 1). Magnetic Resonance Imaging (MRI) of the cervical spine revealed an intermedullary syrinx extending from the second cervical vertebra spine down to the thoracic spine (figure 2), this findings ruled out regional spinal anaesthesia. Based on these findings and after discussion with the patient, an awake fibreoptic intubation for the procedure was planned.

## Anesthetic Management

Topicalisation of the airway was achieved with a combination of nebulised Xylocaine 4% followed by the gargling of topical Xylocaine 4%, which was repeated four times, to ensure absorption by the oropharynx which in this case, was thickened by the deposition of mucopolysaccharide[5]. Finally 2 mls of lidocaine 1% was administered percutaneously into the trachea. The patient was sedated with midazolam 6 milligrams (mg) in 1-2 mg increments and 50 micrograms of fentanyl. The fibreoptic scope was inserted orally into the trachea via a Berman pharyngeal airway, the trachea was intubated with an 7.5 (millimeters internal diameter) endotracheal tube (Mallinckrodt, Athlone Ireland). The head was held in the neutral position during laryngoscopy. Once the endotracheal tube (ETT) was confirmed to be in the correct position by auscultation of breath sounds and by direct visualization of the ETT sitting in the trachea as seen through the fibrebronchocope and Et CO2 monitoring. Anaesthesia was initiated with intravenous (IV) propofol 150 mgs and neuromuscular blockade was obtained using atracurium 50 mgs. An arterial line was inserted into the right radial artery for continuous invasive blood pressure monitoring and blood gas analysis as a means of monitoring the adequacy of ventilation or the development of pulmonary compromise. The patient was positioned on his left lateral side with lateral supports at the pelvis padded with absorbent cotton with gauze. Anaesthesia was subsequently maintained with a combination of an Oxygen/Nitrous Oxide/Sevoflurane mixture and IV Atracurium and Morphine sulphate 10 mg. One liter of Ringer's Lactate solution was infused IV during the case. The case proceeded uneventfully and at the end of the procedure the muscle relaxant was antagonized with IV Neostigmine 2.5 mg along with 0.5 mg IV Glycopyrrolate. The patient resumed normal breathing and, the trachea was extubated awake and in the lateral position without complication. He was then transferred to the post anaesthetic care unit (PACU), where he recovered uneventfully and was monitored overnight. Recovery was uneventful. He is now planning to have the second hip replaced.

## Discussion

Much controversy exists as to whether total hip replacement is best performed under neuraxial block or general anaesthesia (GA)[6]. However in Morquio syndrome the situation is more complex. Anaesthetic implications of Morquio syndrome relate to end-organ dysfunction and anatomical distortions related to the intracellular accumulation of keratin sulfate [7-9]. Difficulty in intubating the trachea is a result of a number of features in particular atlanto-axial instability and hypoplasia of the odontoid process. Additionally, bulky pharyngeal soft tissue due to the deposition of mucopolysaccharides in the soft tissues of the orophayrnx, floor of the mouth, epiglottis, aryepiglottic folds and macroglossia may mandate the use of a smaller endotrachael tube, furthermore the presence of prominent maxillae, limited mouth opening due to involvement of the temporomandibular joints and a short neck makes safe direct laryngoscopy difficult to perform. All of these factors may lead to a "cannot intubate/cannot ventilate" scenario. Furthermore, cervical spine instability in these patients is often not confirmed or excluded by adequate radiographic examination[10] and functional clinical testing and cervical spine stability may not be preserved in the deeply anesthetized patient with neuromuscular blockade.

The complexity of anaesthesia associated with Morquio syndrome has been previously published, particularly in paediatric literature. In a series reporting on occiptio-cervical fusion in 17 patients with Morquio syndrome (age 3-22) intubation under GA is described[11]. Video assisted intubation following gas induction is described in a case series of three pediatric patients undergoing GA for otorhinolaryngology surgery[12]. Furthermore, a case of spinal anaesthesia and a GA with inhalation induction in two children undergoing orthopaedic procedures, and an inhalation induction for a child undergoing stabilisation of cervical spine are described[13,14]. A specifically designed plaster bed which was used to fix the neck during intubation and surgery has been reported[15]. To avoid cervical cord damage in patients with cervical instability, Walker et al described manual in-line stabilisation during intubation[16]. Furthermore, 2 cardiac valve surgery cases under GA is described[17,18]. Nott describes an awake intubation in a patient with unstable neck after spraying the pharynx with topical lignocaine 4% and applying fentanyl and ketamine intravenously[19]. Awake fibreoptic intubations have been previously described in pediatric [20,21] and adult [22] cases of Morquio syndrome. However, the deposition of soft tissue in the neck and oropharynx in Morquio syndrome may present difficulties for conventional fibreoptic intubation, mandating consideration of lighted stylets or fibreoptic stylets[23]. Successful use of a supraglottic airway device as a conduit for fibreoptic -guided tracheal intubation has been described in Hunters syndrome[24]. A preoperative awake fiberoptic examination of the airway by ENT services is advisable.

## Conclusion

There is limited data on anaesthesia in adults with Morquio syndrome, general anaesthesia has been reported in paediatric and to a lesser extent adult patients. Paramount in the anaesthetic care of such patients is a thorough preoperative evaluation of airway in addition to cardiac, respiratory, neurological function.

## Competing interests

The authors declare that they have no competing interests.

## Authors' contributions

AMMcL prepared the manuscript and figures; MF, MD and KF each cared for the patient. All authors have read and approved the final manuscript.

## Pre-publication history

The pre-publication history for this paper can be accessed here:

http://www.biomedcentral.com/1471-2253/10/2/prepub
